# Judicial Opinion 132

**DOI:** 10.1099/ijsem.0.006819

**Published:** 2025-07-07

**Authors:** David R. Arahal, Carolee T. Bull, Henrik Christensen, Maria Chuvochina, Christopher Dunlap, Maria del Carmen Montero-Calasanz, Charles T. Parker, Peter Vandamme, Antonio Ventosa, Stefano Ventura, Peter Young, Markus Göker

**Affiliations:** 1 Departamento de Microbiología y Ecología, Universitat de València, 46010 Valencia, Spain; 2 Department of Plant Pathology and Environmental Microbiology, Pennsylvania State University, 211 Buckhout Lab, University Park, PA 16802, USA; 3 Department of Veterinary and Animal Sciences, University of Copenhagen, Stigbøjlen 4, 1870 Frederiksberg C, Denmark; 4 Leibniz Institute DSMZ – German Collection of Microorganisms and Cell Cultures, Mary-Somerville-Straße 2, D-28359 Bremen, Germany; 5 Crop Bioprotection Research Unit, USDA/ARS/NCAUR, 1815 N. University St, 61604 Peoria, IL, USA; 6 IFAPA Las Torres – Andalusian Institute of Agricultural and Fisheries Research and Training, Cra. Sevilla-Cazalla de la Sierra, 41200, Alcalá del Río, Sevilla, Spain; 7 Department of Energy, Joint Genome Institute, Berkeley, CA 94720, USA; 8 Laboratorium voor Microbiologie, Universiteit Gent, K. L. Ledeganckstraat 35, 9000 Gent, Belgium; 9 Department of Microbiology and Parasitology, Faculty of Pharmacy, University of Sevilla, Sevilla, C/. Prof. Garcia Gonzalez 2, ES-41012 Sevilla, Spain; 10 IRET-CNR, Research Institute on Terrestrial Ecosystems, National Research Council, Via Madonna del Piano 10, 50019 Sesto Fiorentino, Italy; 11 NBFC, National Biodiversity Future Center, Palermo, Italy; 12 Department of Biology, University of York, York YO10 5DD, UK; 13 Leibniz Institute DSMZ – German Collection of Microorganisms and Cell Cultures, Inhoffenstrasse 7B, D-38124 Braunschweig, Germany

**Keywords:** bacteria, corrections, grammar, recommendations, spelling, standards

## Abstract

Opinion 132 deals with a Request for an Opinion asking the Judicial Commission to assess the nomenclatural status of *Skermania piniformis* (Blackall *et al.* 1989) Chun *et al.* 1997, a new combination affected by an unusual change in the epithet of its basonym, *Nocardia pinensis* Blackall *et al.* 1989. This is done by re-evaluating Opinion 85, which sets a precedent. The reasons and consequences of this earlier Opinion are explained in detail, and corresponding guidelines for orthographic corrections are given, along with further guidance derived from more recently published Judicial Opinions. The request to change the epithet to *Skermania pinensis* corrig. (Blackall *et al.* 1989) Chun *et al.* 1997 is granted, and it is also emphasized that this does not affect the legitimacy or the date of valid publication of the names involved. The Judicial Commission also grants the third request made in the same article and rules that, according to Rule 54 of the International Code of Nomenclature of Prokaryotes (ICNP), it is not necessary, and even unwise, to propose two names for the same taxon in the same study to replace an illegitimate name.

## Introduction

In 1997, Chun *et al*. [1,2] proposed the transfer of *Nocardia pinensis* Blackall *et al.* 1989 [3,4] to the new genus *Skermania* Chun *et al.* 1997 as *Skermania piniformis* (Blackall *et al.* 1989) Chun *et al.* 1997. The Request for an Opinion by Li [5] asks the Judicial Commission to assess the nomenclatural status of this name. Is the change of the epithet from *pinensis* to *piniformis* a violation of Rule 41a of the International Code of Nomenclature of Prokaryotes (ICNP) [6]? If so, this would also make *Skermania* Chun *et al.* 1997 an illegitimate name under Rule 20a [6]. Alternatively, is this merely a case of an unwarranted orthographic correction? Then the question would arise as to how the epithet should be spelt.

Li [5] argues that the previous treatment of *Bacteroides forsythus* Tanner *et al.* 1986 [7] vs. *Tannerella forsythensis* (Tanner *et al.* 1986) Sakamoto *et al.* 2002 [8,9] in Judicial Opinion 85 [10] sets an important precedent. We will therefore first assess the consequences of this Opinion and then analyse *S. piniformis* (Blackall *et al.* 1989) Chun *et al.* 1997. We will then address the third question posed by Li [5], namely to clarify the meaning of Rule 54 of the ICNP [6].

## *T. forsythensis* vs. *Tannerella forsythia*

In 2002, Sakamoto *et al*. proposed to transfer the species *B. forsythus* Tanner *et al.* 1986 [7] to the new genus *Tannerella* as * T. forsythensis* [8,9]. The change of the epithet was criticized by Maiden *et al*. [11], who requested the Judicial Commission to correct the epithet to the adjectival form *forsythia*. The request was granted in Judicial Opinion 85 [10].

The etymology given in the effective publication of *B. forsythus* [7] was ‘for.sy’thus. N.L. n. forsythus referring to the Forsyth Dental Center, where the species was first isolated’. The subsequent actions [8,11] raise several questions:

1. Why is *forsythus* in *B. forsythus* interpreted as an adjective [10,11], although the effective publication [7] gives ‘N.L. n.’ (Neolatin noun) as grammar?
2. If it is an adjective, why is *forsythus* orthographically corrected to *forsythia* in *T. forsythia* [10,11] and not just grammatically adapted to *forsytha* in gender?
3. Given that it is an adjective, why is it not acceptable [10,11] to orthographically correct *forsythus* to *forsythensis* [8,9], which is also an adjective?
4. Why is the change to *forsythensis* [8,9] seen as an orthographic correction of *forsythus* that needed to be revised [10,11], rather than the creation of a new epithet?
5. If *forsytha* is corrected to *forsythia* in *T. forsythia* [10,11], does this imply that *forsythus* is also corrected to *forsythius* in * B. forsythus* [7]?

According to Rule 12c of the ICNP [6], an epithet is either a genitive or nominative noun or an adjective (including present and perfect participles). Appendix 9 of the ICNP [6,12,14] gives several examples of how a word from a language other than Latin and Classical Greek can become an epithet. The important thing is that the component must first be Latinized. While the Latinization of words from Classical Greek has its own rules [15], the Latinization of modern language words is usually done by adding one of the endings *-(i)us*, *-(i)a* or *-(i)um*; the choice depends on the type of word. To form a genitive epithet, the singular or plural genitive of the Latinized word must be derived according to the declension of the Latinized word. For example, Appendix 9(G)(4) indicates that for ‘national foods or fermentation products’, the neuter ending *-um* is preferentially added, resulting in a genitive epithet ending in *-i*.

Only a few Latin nouns, such as *quercus*, have a singular genitive that also ends in *-us*. Such less common genitive forms would not normally result from Latinization. The word ‘Forsyth’ in ‘Forsyth Dental Center’ would be Latinized as *forsyth(i)us*, *forsyth(i)a* or *forsyth(i)um* in the nominative case; the genitive case would then be *forsyth(i)i* or *forsyth(i)ae*. An interpretation of the epithet *forsythus* as a genitive noun can therefore be ruled out. However, a nominative in apposition *forsythus* would mean ‘*Bacteroides* the Forsyth’, which is hardly the intended meaning. It makes more sense to interpret *forsythus* as an adjective. Note also that, at least up to and including the 2022 revision of the ICNP [6], the name of a male person called ‘Forsyth’ would be preferentially Latinized as *Forsythius* rather than *Forsythus*, i.e. as a nominative in apposition the epithet would probably also warrant a correction.

To answer the second question, it is necessary to recapitulate how adjectival epithets are formed. To form an adjectival epithet, the stem of the Latinized word must be derived via its singular genitive according to the declension of the Latinized word. A standard Latin adjective ending is then added to the stem. This process is often equivalent to simply adding an adjective ending to a word in a modern language and is therefore presented as such in some parts of the Code [6], such as Appendix 9(E)(2). Recent emendations of Appendix 9(D) of the ICNP [13,16] emphasize more strongly than before the need to Latinize personal names before forming the stem. Standard Latin adjectival endings are listed extensively by Stearn [17]; many of them are used to form the names of taxa above genus rank [18]. The most commonly used standard adjectival ending for epithets is probably *-ensis/-ensis/-ense* for places.

Notably, many of these adjectival endings begin with a vowel, but one vowel is dropped when the adjective is formed from a noun whose stem ends with the same vowel. For example, from the ending *-icus* and L. masc. n. *angelus* (angel; L. gen. masc. n. *angeli*, stem *angel-*), we get L. masc. adj. *angelicus*, while L. fem. n. *Pannonia* (part of Hungary; L. gen. fem. n. *Pannoniae*, stem *Pannoni-*) gives L. masc. adj. *Pannonicus* (not: *Pannoniicus*). Appendix 9 [6] gives some examples of geographical epithets that behave in the same way, e.g. L. masc. adj. *Aegyptius* from *-ius* and L. fem. n. *Aegyptus* (Egypt; L. gen. fem. n. *Aegypti*, stem *Aegypt-*) but L. masc. adj. *Britannicus* from *-icus* and L. fem. n. *Britannia* (Britain; L. gen. fem. n. *Britanniae*, stem *Britanni-*). Other examples show that a connecting consonant may be inserted, or a double vowel, a diphthong, or even a double syllable may be removed [6]. Recent changes to Appendix 9 of the ICNP for more frequent omission of a connecting vowel [12,19] are not directly related, but the avoidance of duplication is a common theme.

If *-us/-a/-um* were a standard adjective ending and Forsyth was Latinized as *forsythus*, *forsytha* or *forsythum*, the adjective would be *forsythus/forsytha/forsythum*. However, *-us/-a/-um* is not a standard adjective ending [17]. When combined with the genitives of either *forsyth(i)us*, *forsyth(i)a* or *forsyth(i)um*, the standard ending *-ius/-ia/-ium* results in *forsythius/forsythia/forsythium*. This still does not explain why this ending was chosen over any of the other standard adjective endings [17]. Standard Latin adjective endings convey slightly different meanings, but the meaning does not seem to have played much of a role in the choice, in accordance with Principle 4 of the ICNP [6]. Since the Forsyth Dental Center can be considered a place, the ending *-ensis/-ensis/-ense* [6] would convey the meaning better than the ending *-ius/-ia/-ium* [17]. However, along with *-eus/-ea/-eum*, which could also have been chosen, *-ius* is the standard adjective ending that is closest in spelling to *-us*. Thus, the epithet in *T. forsythensis* (Tanner *et al.* 1986) Sakamoto *et al.* 2002 was not left unchanged, but corrected to *forsythia* instead of *forsytha*, although *forsytha* is the gender adaptation of the epithet in the basonym *B. forsythus* Tanner *et al.* 1986, when this epithet is interpreted as an adjective.

On the fourth question, while *-ia* was considered a correction of *-a*, the adjectival ending *-ensis* is not a spelling variant of *-ia*, as both are different standard Latin adjectival endings [17] and not particularly close in spelling. Nevertheless, the Judicial Commission did not consider the change from *forsythus* to *forsythensis* (instead of *forsytha*) to be the creation of a new epithet. If this were the case, *T. forsythensis* (Tanner *et al.* 1986) Sakamoto *et al.* 2002 would be an illegitimate name because it would violate Rule 41a [6]. This would also make *Tannerella* Sakamoto *et al.* 2002 an illegitimate name under Rule 20a [6]. Although Judicial Opinion 85 [10] does not explain the issue further, the reason seems to be precisely that *-ensis/-ensis/-ense*, contrary to Maiden *et al*. [11], would better fit the intended meaning of the epithet, i.e. a reference to a place. Sakamoto *et al*. [8,9] may therefore have felt that *forsythus* should have been corrected to the standard Latin adjectival ending that best conveys the meaning, whereas the Judicial Commission [10] felt that the epithet should have been corrected to the standard Latin adjectival ending that was closest in spelling.

The addition of the *-i-* to *forsythia* implies that the *-i-* was already missing in *forsythus*, since the change from the masculine ending *-us* to the feminine ending *-a* is a completely independent adaptation. The epithet *forsythus* is therefore already misspelt, as Maiden *et al*. [11] point out. According to Rule 41a [6], if ‘a species is transferred to another genus without any change of rank, the specific epithet must be retained, except for necessary changes of gender of adjectives used as specific epithets’, unless a *nomen novum* must be formed according to Rule 34a instead of a new combination. So, does changing the gender of adjectives create a new epithet?

Rule 58 [6] implies that an epithet may have different spellings, while Rule 57b defines ‘orthographic variant’ as ‘a name (or epithet) that differs from another name only in the transliteration into Latin … or in its grammatical correctness.’ Note also that section 9 of the ICNP [6] distinguishes between orthographic and grammatical correctness [20]. It follows, e.g. that *forsythus* and *forsytha* are orthographic variants of each other, that *forsythius* and *forsythia* are orthographic variants of each other and that *forsythus* and *forsythius* are not orthographic variants of each other but are still different spellings of the same epithet, as are *forsytha* and *forsythia*. Changing the spelling of an epithet when transferring a species from one genus to another does not change the epithet. The epithet remains the same. Judicial Opinion 105 came to the same conclusion [20].

Therefore, if an orthographic correction is made to the epithet of a homotypic synonym, then that correction is implicitly made to all other homotypic synonyms using that epithet, since the epithet is one and the same. Note that the fact that, for example, at most one of the names *B. forsythus* corrig. and *T. forsythia* corrig. may be considered the correct name by a given user at a given time, while the other must be considered a synonym [21], is not an argument against correcting the spelling of the synonym. First, the ICNP [6] does not specify which of a set of validly published and legitimate names that are considered synonymous and have different positions must be treated as the correct name. Second, even synonyms deserve to be spelt correctly. The call for orthographic and grammatical correctness in, for example, Section 9 of the ICNP [6] is not limited to correct names. This answers the fifth and final question above.

The precedent [10] and the additional considerations lead to the following conclusions.

- The kind of word (adjective, genitive noun or nominative noun) given in the effective publication for a component of a taxon name should be overridden if the overall evidence is against it.
- The fact that the original spelling of a name or epithet does not convey the intended meaning is not a sufficient reason for correcting the spelling.
- If the authors of a new combination change the epithet more than is necessary for a grammatical or orthographic correction, but the link with the original spelling of the epithet is still recognizable and the formation of the changed epithet is better suited to the originally proposed meaning of the epithet, such an action shall be considered as an unwarranted orthographic correction. The epithet should be corrected again without affecting the nomenclatural status of the new combination and the date of its valid publication.
- A non-standard adjectival ending in an adjectival component of a name or epithet derived from a word of a language other than Latin and Classical Greek should be corrected to one of the standard adjectival endings that is closest in spelling, especially if the original spelling of the adjectival component could easily be confused with a noun.
- An orthographic correction of an epithet in the basonym or in a new combination implicitly corrects that epithet in all other homotypic synonyms using that epithet.

Section 9 of the 2022 revision of the ICNP [6] allows spelling corrections to be made by anyone, anywhere [21], except for changes to the first syllable. If there is any uncertainty about the spelling of a name or epithet, the case should still be referred to the Judicial Commission [6]. Authors are therefore advised to follow the above guidelines when making corrections. An overarching theme is to reduce the number of corrections and, if a correction must be made, to reduce the number of differences from the original spelling.

Additional guidance is provided by Judicial Opinions that were published prior to the entry into force of the 2022 revision of the ICNP [6]. Opinion 120 [22] clarified that grammatical corrections (adjustment of the gender of an epithet to the gender of the genus name) should be made with greater zeal than orthographic corrections. Opinion 118 [22] orthographically corrected names that contained misspelt names of persons or of other genera. Opinion 116 [22] emphasized that an incorrect stem of a genus name within a name above genus rank should be orthographically corrected. In conjunction with the 2022 revision of the ICNP [6], we conclude from these Judicial Opinions that such corrections should indeed be made by anyone anywhere, while other types of corrections should be treated with more caution.

It should be noted that the names on the Approved Lists [23] are not special in terms of spelling corrections. While Rule 23a Note 4 [6] emphasizes that the Judicial Commission may correct the Approved Lists, the wording and positioning of this note suggest that it means that the privileges of the Judicial Commission, such as the ability to reject names [24], to correct nomenclatural types [25] and to correct authors of taxon names [26], extend to the Approved Lists despite their special status. Since spelling corrections are no longer a prerogative of the Judicial Commission [6], names from the Approved Lists [23] are subject to the same conditions for spelling corrections as all other names.

## *N. pinensis* vs. *S. piniformis*

The Request for an Opinion by Li [5] suggested that the change of the epithet from *pinensis* to *piniformis* in *S. piniformis* (Blackall *et al.* 1989) Chun *et al.* 1997 [1,2] should not be treated as a violation of Rule 41a of the ICNP [6]. In addition to the precedent discussed above, Li [5] pointed out that the illegitimacy of *S. piniformis* (Blackall *et al.* 1989) Chun *et al.* 1997, which is the name of the type species of *Skermania* Chun *et al.* 1997, would also make the name of that genus illegitimate. The Judicial Commission agrees on the basis of Rule 20a [6]. The suggestion by Li [5] to treat the change of the epithet from *pinensis* to *piniformis* in * S. piniformis* (Blackall *et al.* 1989) Chun *et al.* 1997, merely as a case of an unwarranted orthographic correction, can be evaluated according to guideline (c) above.

The etymology given for *N. pinensis* Blackall *et al.* 1989 [3,4] was ‘M.L. n. *Pinus* genus of pine trees; M.L. adj. *pinensis* pertaining to pines and specifically pine tree like in microscopic morphology’. The word *pinensis* is composed of the stem *pin-* of L. fem. n. *pinus* (L. gen. fem. n. *pini* or *pinus*; stem *pin-*) and the standard Latin adjectival ending *-ensis*, which is used for locations [6]. The word thus formed does not convey the intended meaning of being pine-shaped in the microscope but erroneously indicates a pine tree as the geographical location of the bacterium. According to guideline (c), such an error does not warrant an orthographic correction, but Chun *et al*. [1,2] did just that. The epithet *piniformis* is composed of *pin-*, the connecting vowel *-i-* and the suffix *-formis*, meaning ‘shaped like’, so *piniformis* literally means ‘shaped like a pine tree’. Moreover, the connection between *piniformis* and *pinensis* is still recognizable.

Therefore, according to guideline (c), the change from *pinensis* to *piniformis* should be treated as an unwarranted orthographic correction and the name should be corrected to *Skermania pinensis* corrig. (Blackall *et al.* 1989) Chun *et al.* 1997, without affecting the nomenclatural status and the date of valid publication. This also means that the legitimacy of *Skermania* Chun *et al.* 1997 [1,2] is not affected. Eight commissioners voted in favour of this conclusion, two voted against and two abstained.

## The interpretation of rule 54

Li [5] also asked the Judicial Commission ‘to rule that, according to Rule 54 of the ICNP, there is no need to propose two names for the same taxon in the same study to replace an illegitimate name, as only one of them can be intended to be the correct name.’

Rule 54 [6] states that ‘A name or epithet illegitimate … is replaced by the oldest legitimate name or epithet in a binary or ternary combination that in the new position will be in accordance with the Rules.’ So there is only one intended replacement name and only one position. Proposing multiple names and positions for the same taxon in the same effective publication may actually argue against those names being validly published, according to Rule 28b(1) [6]. The Judicial Commission therefore grants this request. Nine commissioners voted in favour of this conclusion, one voted against and two abstained.

## Abbreviation

ICNP: International Code of Nomenclature of Prokaryotes
